# Nutritional status and associated factors among high school adolescents in Debre Tabor Town, South Gondar Zone, Northcentral Ethiopia

**DOI:** 10.1186/s40795-019-0306-7

**Published:** 2019-11-04

**Authors:** Melkamu Aderajew Zemene, Melaku Tadege Engidaw, Alemayehu Digssie Gebremariam, Desalegn Tesfa Asnakew, Sofonyas Abebaw Tiruneh

**Affiliations:** Public Health Department, College of Health Sciences, Debre Tabor University, P.o. Box: 031, Debre Tabor, Amhara Region Ethiopia

**Keywords:** Adolescent, Nutritional status, High school students, Ethiopia

## Abstract

**Background:**

Adolescents are among the nutritionally vulnerable group due to their nutritional demand for pubertal spurt. At this age, adequate nutrition, nutritional education, and counseling are very important to halt the consequence and its impact on this segment of the population. So, the aim of this study was to assess the prevalence and its associated factors of nutritional status among high school adolescents aged 10–19 years in Debre Tabor Town, South Gondar Zone, and North central Ethiopia.

**Methods:**

A cross-sectional study was conducted from September to October 2017. A total of 349 high school adolescents were selected by using simple random sampling. Data were collected through face to face interview and physical measurement. The data were entered into Epi info version 7 software and then exported into SPSS version 20 software for further analysis. A *p*-value < 0.2 was used to select independent variables for multivariable logistic regression. A p-value ≤0.05 was used to declare the statistical significance. Also, odd ratios were calculated with a 95% of the confidence interval to check the existence of the association.

**Result:**

A total of 327 adolescents participated in this study with the overall response rate of 93.69%. The magnitude of stunting and thinness was 15% (95% CI: 11, 19) and 4.9% (95% CI: 2.4, 7.4) respectively. Sex (AOR: 2.24, 95% CI: 1.15, 4.36), residency (AOR: 2.48, 95% CI: 1.28, 4.82), and family size (AOR: 3.41, 95% CI: 1.65, 7.05) were the associated factors for stunting. Residence (AOR: 3.67, 95% CI: 1.16, 11.56), and living away from the family (AOR: 4.37, 95% CI: 1.20, 15.95) were the associated factors for the development of thinness.

**Conclusion:**

Stunting is a mild public health problem but not thinness. Sociodemographic variables were the determinant factors for the development of stunting and thinness. To halt this, integrated adolescent related school and nutrition services is very important with adequate and quality food access to adolescents. In addition to this the government shall have to increase the access of education.

## Background

Adolescence is a period of life between 10 and 19 years of age with two distinct periods, early (10-14 yrs) and late (15-19 yrs) [1]. As reported in 2012, there were 1.2 billion adolescents worldwide (18%) and 25% of the total population in Ethiopia. At this time the anabolic rate of the body is very high to attain 50% of their adult weight, > 20% of their adult height, and 50% of their adult skeleton [2–4]. Also, growth, maturity and other psychosocial conditions increases their need for macro or/ and micronutrients which leads to malnutrition during a deficiency state [5–7].

Adolescents are the neglected segment of the population related to nutrition and health care services due to major problems in the other vulnerable groups. But adolescents from low and middle-income countries including Ethiopia are a nutritionally vulnerable group with demand of appropriate intervention and monitoring to halt the intergenerational effect of undernutrition. All this shows that there must be more investment on this segment of the population [8–10].

The developmental, economic, social and medical impacts of the global burden of malnutrition are serious and may affect individuals, families, communities and the countries at large [11]. This major public health problem occurs worldwide including South East Asia and Sub- Saharan Africa [9]. In Ethiopia, even if there is economic progress the burden of malnutrition is still very high [12, 13]. Among adolescents malnutrition will lead to retarded physical growth, sexual immaturity, and poor reproductive performance later in life [14] which is reflected in a population as slower growth rate, and short stature [8].

There is little information available about the adolescent age group beside under five children, which is the second most important period of physical growth in life next to infancy [15]. As a study conducted in Mekelle city, Northern Ethiopia, the overall prevalence of obesity, overweight, normal and thinness among adolescents were 0.4, 2, 60.40, and 37.8% respectively [16]. Similarly, a study conducted in Eastern Arsi Zone, Oromia Region, 20.2 and 14.8% of stunting and thinness was observed respectively [17]. In Ethiopia, the sociocultural and environmental backgrounds directly or indirectly affects the nutritional status of adolescents [18]. In addition to this, majority of the students in the study area are living far apart from their parents or families for education. This will lead to inadequate intake due to peer influence, erratic eating habit and absence of family care even if they bring adequate amount of food from the family. So, the aim of this study was to determine the magnitude of stunting and thinness and its determinant factors among high school adolescents to show the burden for policy makers.

## Methods

### Study settings

Debre Tabor Town is the capital of south Gondar Zone administration which is located in Amhara Region, Northcentral Ethiopia. It is 666 km from Addis-Ababa, capital of Ethiopia. It is also in the distance of 100 km southeast of Gondar and 50 km east of Lake Tana. Debre-Tabor town has three high schools; those are Thewodros II, Etegie Tayitu, and Fert. These schools give educational service for the students from Debre Tabor Town and nearby districts (Woredas). Those students came from the woreda were bringing their monthly ration or provision “Sinke in Amharic” from their families to attend their education.

### Study design and period

A cross-sectional study was employed to assess the nutritional status and its associated factors among high school adolescent from September to October 2017.

### Source and study population

All adolescents who were attending high school at Debre Tabor Town are included whereas adolescents with a physical deformity, pregnancy and lactation were excluded from the study.

### Sample size

A single population proportion formula was used to determine the sample size by using 95% confidence level (i.e. 1.96), 5% of marginal error, and 31.5 and 13.6% of proportion for stunting and thinness respectively [19]. The calculated sample size was 332 for stunting and 181 for thinness. Then, we selected the highest sample size and the final sample size after the addition of 5% none response rate was 349.

### Sampling procedures

The list of all students with their names, academic year, respective class and section were obtained from each recorded office of high schools in Debre Tabor Town. The Total numbers of actively engaged students from Etege Tayitu, Feret and Tewodrose II high schools were 787, 1305, and 2297 respectively. Based on this, the calculated sample size was allocated to each school proportionally. Then, we selected 63, 104, and 182 adolescent students from Etege Tayitu, Feret and Tewodrose II high schools respectively by simple random sampling.

### Data collection methods and equipment

The questionnaire was developed by reviewing different studies. A structured face to face interview and physical measurement was done to obtain information regarding a socio-demographic status, health condition, number and types of food items included in their meal, weight and height. We used 24-h recall to determine dietary diversity score at individual level. we considered meals/snacks purchased and consumed both inside and outside at home. Atypical consumption periods during festive periods like national and religious holidays were excluded. The English version questionnaire was translated into the local language, Amharic and was translated back into English to check its consistency by another skilled person. The weights and heights were measured three times by using a weight scale with height stand machine at Frankfurt position, and then the result was recorded to the nearest 0.1 kg and 0.1 cm respectively. Before starting data collection, materials and equipment were always checked. During data collection time, communication between the data collectors, supervisors and the principal investigator were held on a daily basis to update data collection progress and address problems.

### Data quality assurance

A 3 days training with pretest was given for five nurses on the basic skills of height and weight measurement, calibration of instruments, interview techniques, how to obtain written consent or assent and precautions during data collection time. After pretest, corrections were made to the questionnaire. The weight scale has been calibrated by using 1 kg standard weight and the height measurement was checked with other meter tapes. A definition of concepts and terms had been written clearly with the Amharic language to avoid ambiguity. The supervisors and data collectors were recruited outside of the study area to avoid information bias due to familiarization.

### Definitions of terms

**Stunting:** when the adolescent’s height for age was below -2SD as compared to World Health Organization reference point [20].

**Thinness:** when the adolescent BMI-for-age Z score was <−2SD as compared to World Health Organization reference point [20].

**Dietary Diversity Score:** was measured by counting the food items consumed within the previous 24 h and were categorized as poor (consumed < 4 food groups) and good dietary diversity scores (consumed ≥4 food groups). The list of food groups was: 1. Grains, roots or tubers, 2. Vitamin A-rich plant foods, 3. Other fruits or vegetables, 4. Meat, poultry, fish, seafood, 5. Eggs, 6. Pulses/legumes/nuts, 7. Milk and milk products and 8. Foods cooked in oil/fat. All this were coded as 0 if not consumed within the past 24 h and 1 if consumed [21].

**‘Teff”:** an African cereal which is cultivated almost exclusively in Ethiopia, used mainly to make flour.

### Data processing and analysis

The collected data were checked for completeness and consistency by the supervisors and the principal investigator during and after data collection. The data was managed by editing, verification, coding, classification, and tabulation during data entry and analysis. Data were entered by using Epi info 7 and Anthroplus software. Then these data were transported into SPSS version 20 statistical software for descriptive and analytical analysis. Descriptive analysis was carried out to describe the variables in number and proportion and the analytical analysis were carried out to see the crude and adjusted effect of each variable. A *p*-value of less than 0.2 was used to select candidate variables for multivariable logistic regression.

In this study, a p-value of less than or equal to 5% was considered as statistically significant after fitting into multivariable logistic regression models and odd ratios were calculated with a 95% of confidence interval had been used at this stage to assess the independent and multivariable effect. The Hosmer-Lemeshow goodness of fit test was performed to assess how well the constructed model was good.

A binary logistic regression was done to assess the existence of an association between independent variables and the stunting of high school adolescent students. From these explanatory variables; sex, marital status, place of residence and family size were associated with stunting. After, these variables were taken into multivariate logistic regression; sex, residence and family size were found to be significant factors for stunting. Similarly, Bivariate analysis was done to assess the existence of an association between independent variables and the thinness of high school adolescent students. Thus, sex, residence, grade, living status, and source of drinking water were found to be associated factors with the thinness of adolescent students. After fitting these variables into a multivariable logistic regression, only residence and living status were found to be significant factors for thinness.

## Result

### Socio-demography characteristics

In this study, a total of 327 school adolescences were included with a response rate of 93.69%. The mean ± Standard Deviation (SD) of age was 17.54 ± 1.23 years and all of them were found in the late age groups (15-19 yrs). Most of (93.6%) the adolescents were single in marital status. About two-thirds (65.1%) and 34.9% of respondents’ residence were urban and rural respectively as shown in the table below (Table 1).

**Table 1:** Socio-demographic characteristics of high school adolescent students in Debre Tabor Town, South Gondar Zone, Northcentral Ethiopia, 2017 (*N* = 327)

Variables	Categories	Number	Percentage (%)
Sex	Male	160	48.9
	Female	167	51.1
Age	15–19	327	100
Marital status	Single	306	93.6
	Married	21	6.4
Residence	Urban	213	65.1
	Rural	114	34.9
Grade	9th	84	25.7
	10th	243	74.3
Religion	Orthodox	313	95.7
	Muslim	4	1.2
	Protestant	10	3.1
Living status	Away from family	132	40.4
	Within family	195	59.6
Educational status of head of the family	Elementary	104	31.8
	High school	38	11.6
	College	10	3.1
	University	29	8.9
	No Education	146	44.6
Occupation of head of the family	Daily laborer	7	2.1
	Farmer	232	70.9
	Merchant	29	8.9
	Public servant	28	8.6
	Others*	31	9.5
Dietary Diversity	< 4 food items	166	50.8
Score/ DDS	≥4 food items	161	49.2

Others* = housewife, unemployed, retired from work)

### Dietary diversity

About 184(56.3%) of participants were using “teff” as a staple diet. Whereas, 132(40.4%) of the respondents were using “teff”, barely, wheat, and maize in combinations as their staple diet. From all, 161(49.2%), and 166(50.8%) of participants had good, and poor dietary diversity scores respectively.

### Nutritional status of high school adolescent students

Weight, height, sex, and age were used to determine the nutritional status of the respondents. The mean ± SD of weight and height of male respondents were 54.27 kg ± 5.81 kg, 167.27 cm ± 7.94 cm respectively whereas 48.97 kg ± 6.76 kg, 156.44 cm ± 6.35 cm respectively were for females.

The average ± SD of respondents’ Body Mass Index (BMI) was 19.69 ± 2.24 kg/m^2^. According to WHO classification, 16(4.9%) of participants had moderate underweight with respective to their BMI. But none of the participants were classified as overweight and obese.

Based on WHO growth reference curve of height for age (stunting), 15% (95% CI: 11, 19) of the participants were stunted. Among these, 12(3.7%) of participants were < −3SD and 37(11.3%) of participants were < −2SD were severely and moderately stunted respectively at a mean ± standard deviation of − 1.03 ± 0.98.

The overall thinness rate as compared to WHO growth reference curve of BMI for age was 4.9% (95% CI: 2.4, 7.4) and all of the participants were below -2SD (moderate). Similarly, 4.9% of participants were found < +1SD with a mean ± SD of − 0.72 ± 0.89. The distribution of BMI for age of the respondents were similarly 4.9% with respective of <−2SD and < +1SD.

### Factors associated with stunting and thinness

#### Associated factors of stunting

Female high school adolescent students were 2.24 times (AOR: 2.24, 95% CI: 1.15, 4.36) more likely to develop stunting as compared to males. Adolescent students who came from rural areas were 2.48 times (AOR: 2.48, 95% CI: 1.28, 4.82) more likely to develop stunting as compared to those who were from the urban area. Adolescent students who were living from a household with a family size of six and above were 3.41 times (AOR: 3.41, 95% CI: 1.65, 7.05) more likely to develop stunting as compared with adolescents who were from a household with a family size of less than six members as shown in the table below (Table 2).

**Table 2:** logistic regression result of associated factors with stunting and thinness among high school adolescent students in Debre Tabor Town, South Gondar Zone, Northcentral Ethiopia, 2017

Logistic regression results for stunting					
Variables	Category	Stunting		COR (95% CI)	AOR (95% CI)
		Yes	No		
Sex	Male	17	143	1	1
	Female	32	135	1.99 (1.05, 3.75)	2.24 (1.15, 4.36) *
Marital status	Single	43	263	1	1
	Married	6	15	2.44 (0.90, 6.65)	2.31 (0.81, 6.62)
Residence	Urban	25	188	1	1
	Rural	24	90	2.00 (1.08, 3.70)	2.48 (1.28, 4.82) *
Family size (mean)	< 6	13	126	1	1
	≥6	36	152	2.29 (1.16, 4.51)	3.41 (1.65, 7.05) *
Logistic regression results for thinness					
Variables	Category	Thinness		COR (95% CI)	AOR (95% CI)
		Yes	No		
Sex	Male	5	155	1	1
	Female	11	156	2.18 (0.74, 6.43)	1.82 (0.51, 6.53)
Residence	Urban	5	208	1	1
	Rural	11	103	4.44 (1.50, 13.12)	3.67 (1.16, 11.56) *
Grade	9th	9	77	1	1
	10th	7	234	0.42 (0.15, 1.17)	0.27 (0.06, 1.08)
Living status	Away from family	10	122	2.58 (0.91, 7.28)	4.37 (1.20, 15.95) *
	Within family	6	189	1	1
Drinking water Source	Protected	5	168	1	1
	Unprotected	11	143	2.58 (0.87, 7.61)	2.86 (0.91, 9.02)

*COR* Crude Odd Ratio, *AOR* Adjusted Odd Ratio, *CI* Confidence Interval

* significant at *p*-value ≤0.05, 1 = reference

#### Associated factors of thinness

Adolescent students who came from rural areas were 3.67 times (AOR: 3.67, 95% CI: 1.16, 11.56) more likely to develop thinness as compared to those who were from the urban area. Adolescent students who were living away from their respective family with a monthly allowance/subsistence were 4.37 times (AOR: 4.37, 95% CI: 1.20, 15.95) more likely to develop thinness as compared to those who were living along with their respective family as shown in the table below (Table 2).

## Discussion

Malnutrition remains among the most devastating problems and nearly 30% of people worldwide are suffering from one or more of the multiple forms of malnutrition. Especially these days, undernutrition among adolescents is a serious public health problem particularly in developing countries [11]. However, previous studies in Ethiopia didn’t show the nutritional status of adolescents at the country level. So, this study was conducted to determine the magnitude and associated factors of nutritional status among high school adolescents.

The overall prevalence of stunting was found to be 15% (95% CI: 11, 19) which is a mild public health problem. This is in line with a study conducted in Mieso woreda, Somalia regional state, Ethiopia, which was 11.5% [18]. The magnitude was high as compared with 7.2%, reported from Chiro Town, West Hararge, Ethiopia [22]. But it was low as compared with a study done in Aseko District, Eastern Arsi Zone which was 20.2% [17]. Since stunting is chronic malnutrition, this difference could be due to the differences in socioeconomic status, seasonal variation, educational status of the families regarding nutrition and food culture. The overall prevalence of thinness was 4.9% (95% CI: 2.4, 7.4) which is much lower than a result reported from a study done in Mekelle city, Northern Ethiopia which was 37.8% [16]. The prevalence of thinness in this study was also compared with a study done in Mieso woreda, Somalia region, and the result was 22.9% (95%, CI: 19.7, 26.1) which is high as compared with this study. The possible reason for this difference may be the seasonal variations in the data collection period, socioeconomic status, and geographical areas with dietary habit difference.

Female adolescent students were 2.24 times (AOR: 2.24, 95% CI: 1.15, 4.36) more likely to be stunted as compared to male which is in line with a study conducted in West Bengal, India [23] and in Mieso woreda, Somalia region [18]. On the other hand, this result is not in line with the study result from Tehuledere District, Northeast Ethiopia, where males were 2.4 times stunted than females [24]. This variation may be in part due to socioeconomic difference, gender preference, and care delivered due to this difference. The other possible reason may be eating disorder of adolescents girls like repeat skipping of meals which leads inadequate dietary intake [17].

Adolescent students who came from rural areas were 2.48 times (AOR: 2.48, 95% CI: 1.28, 4.82) more likely to be stunted as compared to those who were from the urban area. This finding is supported by a study done in Mizan District, South Western Ethiopia [25]. Possible reasons for this may be environmental opportunities of urban families far differ from rural inhabitants like family networks to access health care and other services, a high number of family size, literacy, and children highly engaged in field works. In this study, the other factor significantly associated with being stunted was family size. Adolescents who were from a household with a family size of six and above were 3.41 times (AOR: 3.41, 95% CI: 1.65, 7.05) more likely to be stunted as compared with adolescents who were from a household with a family size of less than six. This finding was in line with a result reported from Adwa Town, North Ethiopia [26]. This may be explained by households of large family members with low socioeconomic status and birth interval and lack of awareness on feeding practices and nutritional requirement of the children. All this will lead to sharing food when the household has insufficient food which will compromise the adequate intake of food at an individual’s level.

In this study, Adolescents who came from rural areas were 3.67 times (AOR: 3.67, 95% CI: 1.16, 11.56) more likely to develop thinness as compared to those who were from the urban area. This finding is in line with a study done in Dehradun district, India, where the prevalence of thinness were 25.9 and 6.1% in rural and urban respectively [27]. Again, this result was in line with a study done in Mizan District, South Western Ethiopia, where both thinness and stunting were higher in rural areas [25]. The possible reasons for this may be that urban dwellers have better access to safe water and sanitation, sanitary toilet facilities and health care services than rural adolescents [28].

Adolescent students who were living away from their respective family were 4.37 times (AOR: 4.37, 95% CI: 1.20, 15.95) more likely to be thinner than those who were living along with their family. The possible explanation may be due to inadequate intake because of monthly subsistence, change in behavior, and inadequate allocation of monthly ration or subsistence.

One of the major strengths of the study was random selection of schools and adolescents. So, generalization to all adolescents in the study area is possible since an attempt was made to identify randomized schools and adolescents. A limitation was the use of cross sectional study design which could only generate a hypothesis regarding the role of independent variables on the nutritional status of high school students but not their cause and effect relationships.

## Conclusion

In this study, stunting is a mild public health problem but thinness is not a public health significant problem. The sex, being from the rural area, and family size were the determinant factors for the development of stunting. The associated factors for the development of thinness were residence area and living away from their respective family of the respondents.

So, appropriate counseling and health education based on sex, family size, and living conditions of the respondents are very important since this is a critical age to have peer influence, erratic eating behavior and psychosocial problem among females due to starting of menstruation. In addition to this, the government have to increase the access of schools in the rural parts of the country to reduce the burden living away from the family and to increase the family care towards adolescents.

## Data Availability

The datasets are available from MTE. So, he can provide the data at any time during the request.

## Abbreviations

BMI: Body Mass Index
MoE: Ministry of Education
MoH: Ministry of Health
SPSS: Statistical package for social science
UNICEF: United Nation Children’s Education and Fund
WHO: World Health Organization
